# The development and cross-national validation of the short health literacy for school-aged children (HLSAC-5) instrument

**DOI:** 10.1038/s41598-023-45606-1

**Published:** 2023-10-31

**Authors:** Olli Paakkari, Markus Kulmala, Nelli Lyyra, Minna Torppa, Joanna Mazur, Zuzana Boberova, Leena Paakkari

**Affiliations:** 1 Research Centre for Health Promotion, Faculty of Sport and Health Sciences, University of Jyväskylä, Jyvaskyla, Finland; 2 Department of Teacher Education, Faculty of Education and Psychology, University of Jyväskylä, Jyvaskyla, Finland; 3 Department of Humanization in Medicine and Sexology, Institute of Health Science, University of Zielona Gora, Zielona Gora, Poland; 4 Institute of Biology and Ecology, Faculty of Science, Pavol Jozef Šafarik University in Košice, Kosice, Slovakia

**Keywords:** Health policy, Public health

## Abstract

Health literacy is an asset for and indicator of adolescents’ health and wellbeing, and should therefore be monitored and addressed across countries. This study aimed to develop and validate a shorter version of the original 10-item health literacy for school-aged children instrument in a cross-national context, using data from the health behaviour in school-aged children 2017/18 survey. The data were obtained from 25 425 adolescents (aged 13 and 15 years) from seven European countries. Determination was made of the best item combination to form a shorter version of the health literacy instrument. Thereafter, the structural validity, reliability, measurement invariance, and criterion validity of the new 5-item instrument were examined. Confirmatory factor analysis showed a good model fit to the data across countries and in the total sample, confirming the structural validity (CFI = 0.995, TLI = 0.989, SRMR = 0.011, RMSEA = 0.031). The internal consistency of the instrument was at a good level across countries (α = 0.87–0.98), indicating that the instrument provided reliable scores. Configural and metric invariance was established across genders, ages, and countries. Scalar invariance was achieved for age and gender groups, but not between countries. This indicated that the factor structure of the scale was similar, but that there were differences between the countries in health literacy levels. Regarding criterion validity, structural equation modelling showed a positive association between health literacy and self-rated health in all the participating countries. The new instrument was found to be valid and reliable for the purposes of measuring health literacy among adolescents in a cross-national context.

## Introduction

Different types of skills and knowledge have been identified as central components of wellbeing^1^. One such component is health literacy. Health literacy is a set of competencies that are required to promote and sustain one’s own health and that of others^2,3^, bearing in mind the need for people to ‘take control of their health and lead fulfilling lives with a sense of meaning and purpose, in harmony with nature’^4^. According to WHO^5^, all adolescents need ‘a broad range of health-related skills and competencies, including skills needed to navigate in virtual environments and digital contexts; the ability to make critical judgments about health and appraise the reliability of health messages across different communication channels; to be able to act in an ethically responsible way in health matters within a diverse and changing world; to become aware of their own needs, perceptions, wishes, and preferences in relation to physical, social and mental health and well-being; and to be able to participate in influencing and carrying out decisions and actions that impact their health, that of others, the environment, and wider society’.

Health literacy is both an asset for and an indicator of health and wellbeing, including among adolescents, and it should therefore be monitored and addressed internationally^5^. The monitoring of health literacy supports national and regional evidence-informed policy and practice related to the promotion of adolescents’ health and wellbeing.

Among both adults and adolescents, health literacy has been found to explain the variance in health outcomes. Since health literacy competencies can be learned and developed, the disparities in health outcomes caused by health literacy differences can be regarded as avoidable health inequalities, such that disparities in health literacy constitute a health equity issue. Among adolescents, higher health literacy has been linked to many favourable health indicators, including higher levels of physical activity^6–9^, self-rated health^8,10^, better sleeping^8^, and better eating habits^6,8,11^. Furthermore, it has been associated with lower levels of smoking^8,12,13^, alcohol use^8,12^, health complaints^8^, problematic social media use^10^, and obesity^13,14^. This strand of research has mainly focused on measuring health literacy as a perceived (i.e. subjective) evaluation of one’s competencies. According to the meta-analysis of Sheeran et al.^15^, perception of one’s own competence is an important factor, requiring attention in interventions to change health behaviour. Furthermore, among adolescents, perceived health literacy has been shown to correlate well with measured health literacy^16^. A similar link between perceived and measured competencies has been found in several PISA studies, such as those on digital reading^17^ and mathematics^18^.

Research on the link between health literacy and health has often focused on selected health indicators, and only rarely on a broad spectrum of indicators. Furthermore, to our knowledge, there has been no longitudinal research on how individual differences in health literacy develop, or on the underlying environmental and individual factors that might explain these differences. To measure health literacy in association with a comprehensive set of health indicators and/or environmental and individual factors, it will be necessary to have health literacy instruments that are brief enough to be integrated within longer surveys or broader study designs. Furthermore, the need to shorten the health literacy instrument has emerged from observations in everyday research. Limitations in the concentration and perseverance of children and adolescents, together with the need to answer a questionnaire that measures several different areas at the same time, can cause fatigue and lack of interest in young respondents, thus reducing the reliability of the survey^19^.

The monitoring of health literacy across countries calls for theoretically-sound measures that are internationally validated, preferably with the same statistical analyses and sample characteristics^20^. These premises served as a starting point for developing the health literacy for school-aged children (HLSAC) scale^21^ in the context of the international health behaviour in school-aged children (HBSC) study. The characteristics of health literacy, as measured by HLSAC, are that it is subjective (involving adolescents’ own evaluation of their competencies), comprehensive, and general (i.e. not focused on one particular health topic). The HLSAC scale contains 10 items that focus on five theory-based components identified as constituting health literacy, as follows: (1) theoretical health information (having knowledge on health-related topics), (2) practical health-related skills (the ability to put theory into practice, e.g. health information-seeking skills, hygiene-related skills), (3) critical thinking skills, (4) self-awareness (i.e. the ability to reflect on issues from one’s personal perspective), and (5) citizenship (i.e. the ability to think of the consequences of one’s actions for the environment, and to promote health in one’s surroundings)^3^.

Cross-national comparative analysis has shown that the 10-item HLSAC instrument is useful in identifying disparities in health literacy within and between countries^22^. Furthermore, the instrument has worked well in detecting the link between health literacy and health^8,9,11,12^. In this context, there has also been discussion on the importance of health literacy as a determinant of health. The discussion has encompassed its power to estimate health outcomes with reference to self-rated health^22^. According to Idler and Benyamini^23^, one’s assessment of ‘How in general would you rate your health?’ as an item is ‘a most powerful self-assessment, combining myriad factors from many different domains of life’. In fact, self-rated health has been found to be a robust indicator to predict mortality^24^ and research has shown an association between better self-rated health and higher health literacy^25–28^. Similar findings have been found with the HLSAC instrument when used among adolescents^8,22^.

The HLSAC scale has been shown to have good potential to measure health-related skills and knowledge in international contexts, and to explain the variance in health outcomes. Though HLSAC is shorter than many other health literacy instruments^29^, an even shorter version could be useful, bearing in mind the considerable length of many comprehensive health surveys.

Previous research has shown that longer versions of unidimensional measures can be reduced to a third of their original length^30^. This led us to assume that HLSAC, as a one-factorial measure, could be shortened by half, while bearing in mind the intention of Appel et al.^31^, ‘to identify a short form with a substantially reduced number of items, which nonetheless exhibits levels of reliability and validity that are comparable to the long form’ (p. 419). In seeking to test the correspondences of the reliability and validity of the original scale with those of the shortened version, the overall test procedures should be the same. In shortening the HLSAC, this means testing the combination of health literacy items that would best predict the original HLSAC instrument, while addressing also the instrument’s structural validity and model fit, its reliability coefficient and internal consistency, and its measurement invariance. In addition, to compare the criterion validity of the scales at different lengths, it is necessary to test whether health literacy, as measured with a shorter version, is linked to other phenomena—including self-rated health^22^—in the same manner as the longer version^20^.

There is no gold-standard method for reducing the number of items in a measure. In fact, a variety of approaches may be utilized, combining both theoretical and data-driven methodologies.

### Aim of the study

This study aimed to develop and validate a short (5-item) version of the original 10-item HLSAC instrument in a cross-national context (Finland, Estonia, Poland, Czechia, Belgium (Fl.), Slovakia, Germany), using nationally representative data from the HBSC study. In pursuit of this aim, we first determined the best possible combination of items to form a shorter version of the HLSAC scale, taking one item from each of the five theory-based components. We also provided instructions for classification into low, moderate, and high health literacy levels. We then examined the structural validity, reliability, measurement invariance, and criterion validity of the new instrument, here referred to as HLSAC-5.

## Results

### Construction of the HLSAC-5 instrument

Firstly, regression analyses were conducted to identify the best set of five items for the short health literacy scale. The 10-item score was predicted via a 5-item score consisting of five health literacy items, constrained in such a way as to have at least one item from each core component of health literacy (Table 1). Regression analyses were conducted separately for each country and for all the countries together; this yielded a total of 32 models for each of these conditions.

**Table 1:** Theoretical components of health literacy and corresponding HLSAC items (10 items in HLSAC, five items in HLSAC-5).

Theoretical component		Item *I am sure that…*
Theoretical knowledge	**HL1** HL5	**I have good information about health** I can easily give examples of ways or things that promote health
Practical knowledge	**HL7** HL4	**When necessary, I find health-related information that is easy for me to understand** I can follow the instructions given to me by healthcare personnel (e.g. nurse, doctor)
Critical thinking	**HL3** HL9	**I can compare health-related information from different sources** I can usually figure out if some health information is right or wrong
Self-awareness	**HL10** HL8	**I can give reasons for choices I make regarding my health** I can judge how my own behaviour affects my health
Citizenship	**HL6** HL2	**I can judge how my own actions affect the surrounding natural environment** When necessary, I am able to give ideas on how to improve health in my immediate surroundings (e.g. a nearby place or area, family, friends)

The five items selected for HLSAC-5 are marked in bold.

Table 2 reports the adjusted amount of explained variance (R^2^) in the country-specific and combined analyses for the 5-item instrument, in terms of predicting the original HLSAC instrument. The results show that while variation in explanatory power exists between countries, it is relatively limited. Although the best combination of health literacy items in the total sample (items HL1, HL3, HL6, HL7, and HL10) did not rank highest in every country when the countries were analysed separately, it still performed very close to the country-specific best combination. The largest difference between the country-specific best item set (explained variance) and the best total sample item set (explained variance) was 0.0153 (Belgium (Fl.)), and the smallest 0.0007 (Slovakia). Taking into account the minimal variability in explanatory power between the total sample and the best country set of items in five countries and the consistent results in two countries, the combination of HL items HL1, HL3, HL6, HL7, and HL10 was regarded as providing the best combination of items to form the short HLSAC-5 instrument (Tables 1 and 2).

**Table 2:** Adjusted amount of explained variance for the best 5-item HLSAC combinations in predicting the original 10-item HLSAC instrument.

Country	Total sample	Finland	Estonia	Poland	Czechia	Belgium (Fl.)	Slovakia	Germany
Best HL items	HL1, HL3, HL6, HL7, HL10	HL1, HL3, HL6, HL7, HL10	HL5, HL3, HL6, HL7, HL10	HL5, HL3, HL6, HL7, HL10	HL1, HL3, HL6, HL7, HL10	HL5, HL3, HL6, HL7, HL10	HL2, HL5, HL7, HL9, HL10	HL2, HL5, HL7, HL8, HL9
R^2^ adjusted for country-specific best items	0.9027	0.9624	0.9048	0.8747	0.9225	0.8683	0.8776	0.8920
R^2^ adjusted for best 5 items (HL1, HL3, HL6, HL7, HL10)	0.9027	0.9624	0.8985	0.8685	0.9225	0.8530	0.8769	0.8894
Range of R^2^ adjusted values for all eligible combinations	[0.8703, 0.9027]	[0.9418, 0.9624]	[0.8669, 0.9048]	[0.8302, 0.8747]	[0.8893, 0.9225]	[0.8248, 0.8683]	[0.8359, 0.8776]	[0.8434, 0.8920]

**Figure 1:**
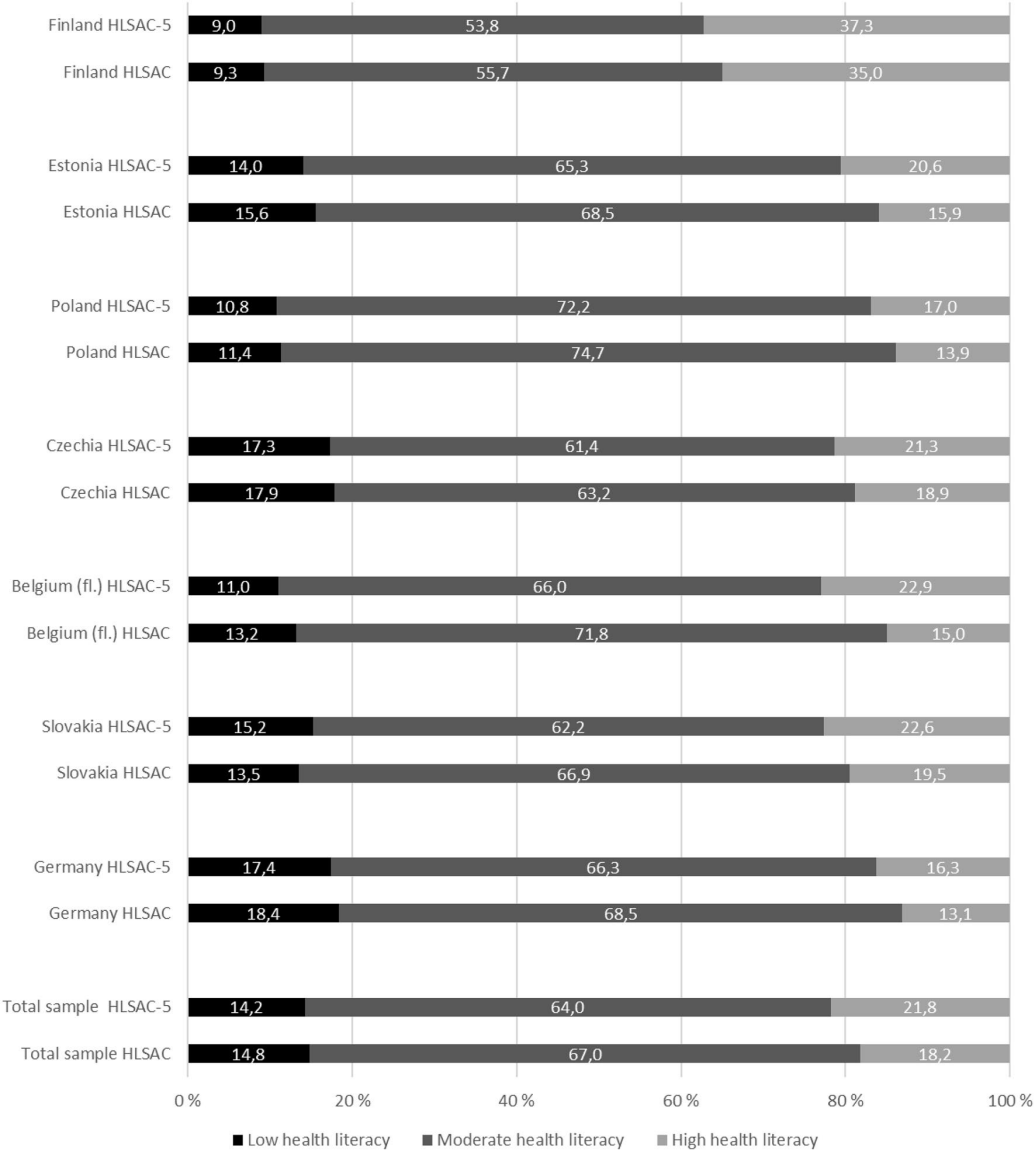
Levels of subjective health literacy as measured with HLSAC-5 and HLSAC; by country, and for the total sample (percentage distribution).

When the instruments were compared, the distribution analysis showed that the short HLSAC-5 performed logically, and in the same way as the original HLSAC instrument (Fig. 1). When measured with the short HLSAC-5 instrument, the number of respondents with low health literacy was systematically slightly lower (range 0.3–2.2 percentage points) in all the countries except Slovakia, as compared to the results obtained with the original HLSAC instrument. In addition, when measured by the HLSAC-5 instrument, the number of respondents with moderate health literacy decreased slightly (range 1.8–5.8 percentage points), and the number of respondents with high health literacy increased (range 2.3–7.9 percentage points) in all the countries as compared to the results obtained with the original HLSAC measure. Across the total sample, the differences between the health literacy levels were small (low = 0.6, moderate = 3.0, high = 3.6 percentage points), as measured by the short and the original HLSAC instrument.

### Structural validity, item validity, and reliability of the HLSAC-5 instrument

Means, variances, and correlations for the five health literacy items included in HLSAC-5 are presented in Table 3. All the health literacy items were moderately correlated (with correlations varying between 0.36 and 0.48).

**Table 3:** Spearman correlation coefficients between the items in the HLSAC-5; also item means, standard deviations, skewnesses, and kurtoses for the total sample.

	HL1	HL3	HL6	HL7	HL10
HL1	1.00				
HL3	0.40***	1.00			
HL6	0.36***	0.40***	1.00		
HL7	0.38***	0.48***	0.47***	1.00	
HL10	0.40***	0.43***	0.45***	0.47***	1.00
Mean	3.19	2.84	3.09	3.08	3.03
Standard deviation	0.74	0.80	0.79	0.78	0.77
Skewness	− 0.75	− 0.40	− 0.64	− 0.62	− 0.56
Kurtosis	0.50	− 0.20	0.01	0.11	0.06

***Spearman’s rank correlation significant at the 0.001 level.

CFAs were conducted in order to confirm the structural validity of the HLSAC-5 (consisting of five items selected on the basis of the regression analyses described above). In the CFA, the five items were set to load on one factor. The CFA models were run separately for each country. The results showed that in the total sample and in all the countries, a one-factor model had a good model fit (min. CFI and TLI = 0.979 and 0.958; max. RMSEA and SRMR = 0.047 and 0.020) (Table 4).

**Table 4:** Model fit indices for the total sample and for each country.

	Total sample	Finland	Estonia	Poland	Czechia	Belgium (Fl.)	Slovakia	Germany
Χ^2^(5)	119.052	28.462	20.047	43.173	19.454	19.725	19.16	22.617
*p* value	< 0.001	< 0.001	0.001	< 0.001	0.002	0.001	0.002	< 0.001
CFI	0.995	0.993	0.994	0.979	0.998	0.990	0.993	0.992
TLI	0.989	0.987	0.989	0.958	0.996	0.981	0.987	0.985
RMSEA	0.031	0.047	0.031	0.047	0.020	0.033	0.030	0.035
SRMR	0.011	0.011	0.012	0.020	0.008	0.014	0.013	0.014

The *p* value is calculated from the Chi-square goodness-of-fit test.

In the total sample, standardized factor loadings ranged from 0.59 to 0.72. Finland had the highest factor loadings (λ = 0.79–0.87) and Poland the lowest (λ = 0.48–0.64) (Table 5). The lowest standardized factor loading, 0.48, was in Poland, for item HL1 (‘I have good information about health’). Item-level reliability coefficients (R^2^) ranged from 0.23 (Poland, Item HL1: ‘I have good information about health’) to 0.76 (Finland, Item HL7: ‘I find health-related information that is easy for me to understand’), suggesting that the latent factor explained 23–76% of the item variances. Cronbach α values for the five items ranged from 0.87 to 0.98, indicating a high internal consistency for the 5-item instrument in all countries.

**Table 5:** Standardized factor loadings, reliability coefficients (R^2^, in parenthesis), and Cronbach α values of HLSAC-5.

	Total sample λ (R^2^)	Finland λ (R^2^)	Estonia λ (R^2^)	Poland λ (R^2^)	Czechia λ (R^2^)	Belgium (Fl.) λ (R^2^)	Slovakia λ (R^2^)	Germany λ (R^2^)
HL1	0.59 (0.35)	0.79 (0.63)	0.63 (0.39)	0.48 (0.23)	0.61 (0.37)	0.52 (0.28)	0.53 (0.28)	0.57 (0.32)
HL3	0.67 (0.45)	0.85 (0.71)	0.70 (0.48)	0.59 (0.35)	0.71(0.50)	0.54 (0.29)	0.66 (0.44)	0.62 (0.39)
HL6	0.65 (0.43)	0.83 (0.68)	0.59 (0.35)	0.58 (0.34)	0.73 (0.54)	0.50 (0.25)	0.59 (0.35)	0.62 (0.38)
HL7	0.72 (0.52)	0.87 (0.76)	0.69 (0.48)	0.64 (0.40)	0.77 (0.59)	0.67 (0.44)	0.64 (0.41)	0.72 (52)
HL10	0.68 (0.46)	0.83 (0.68)	0.62 (0.38)	0.58 (33)	0.76 (0.57)	0.64 (0.41)	0.64 (0.41)	0.63 (0.39)
α	0.80	0.98	0.95	0.93	0.97	0.87	0.95	0.93

### Measurement invariance of the HLSAC-5 instrument across genders, ages, and countries

The analysis of configural and metric invariance showed that constraining the factor loadings to be equal across countries, age groups, and genders did not substantially decrease the model fit (Country invariance: ΔCFI = 0.001, ΔRMSEA = 0.005; Age invariance: ΔCFI = 0.000, ΔRMSEA = 0.002; Gender invariance: ΔCFI = 0.000, ΔRMSEA = 0.001), indicating that the factor structure was comparable across countries, age groups, and genders (Table 6).

**Table 6:** Measurement invariance by country, age, and gender. Results of configural, metric, and scalar invariance.

	Model fit				Change in model fit	
	Χ^2^	df	CFI	RMSEA	ΔCFI	ΔRMSEA
Country invariance						
Configural	51.336	35	0.999	0.012		
Metric	111.655	59	0.998	0.016	0.001	0.005
Scalar	705.602	83	0.978	0.048	0.020	0.031
Age invariance						
Configural	37.327	10	0.999	0.015		
Metric	44.413	14	0.999	0.014	0.000	0.002
Scalar	116.695	18	0.997	0.022	0.002	0.008
Gender invariance						
Configural	36.210	10	0.999	0.015		
Metric	48.235	14	0.999	0.015	0.000	0.001
Scalar	157.205	18	0.995	0.026	0.004	0.011

In assessing for scalar invariance, the intercepts of the five items were constrained to be equal across all groups. ΔCFI and ΔRMSEA values showed scalar invariance for age and gender, but not perfectly for country. These results indicated that for both age groups and both genders, the factor structure and item levels did not differ. However, there were differences between countries, indicating that while the factor structure was similar, there were health literacy level differences between the countries (Table 6).

### Criterion validity

**Figure 2:**
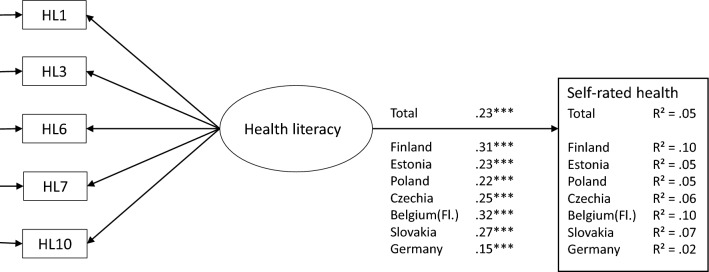
Regression coefficients of health literacy explaining the variance in self-rated health in total and by country. Factor loading set to be equal, standardized beta coefficient estimates reported. ***p < 0.001.

In the total/pooled sample, there was a positive association between health literacy and self-rated health, indicating that higher health literacy was associated with higher self-rated health. In line with the pooled sample, the analysis by country showed a positive association between health literacy and self-rated health in all seven participating countries, with regression coefficients ranging from β = 0.15 in Germany to β = 0.32 in Belgium (Fl.). Health literacy explained self-rated health to some extent, ranging from 2% in Germany to 10% in Belgium (Fl.) and Finland (Fig. 2).

## Discussion

The aim was to construct and validate a brief, theoretically comprehensive, and internationally comparable instrument for measuring children’s and adolescents' subjective health literacy. The cross-national development and validation process encompassed seven European countries and was based on the original 10-item HLSAC instrument^21^. The original HLSAC instrument has been validated in several studies^9,32–41^ and has proven to be an appropriate tool for measuring subjective health literacy. However, for large-scale studies where the purpose is to explore the relationship between health literacy and other phenomena, there is a need for shorter instruments.

The research resulted in a 5-item HLSAC-5 instrument containing one item from each of the health literacy theoretical core components. For the total sample, the HLSAC-5 instrument had good predictive properties, predicting 90% of the variance of the original 10-item HLSAC instrument. The HLSAC-5 was constructed as a one-factor model, similar to the original longer HLSAC instrument. All five items were derived from a conceptual framework; they contribute substantially to the underlying construct of health literacy, addressing the recommended attributes for such a measure^42,43^. The essential advantage of the one-factor model is that it does not violate the requirement on additivity, and enables reliable calculation and interpretation of sum scores. It thus differs from multifactorial health literacy scales, which consist of several subscales^44,45^. Although the HLSAC-5 instrument is based on five theoretical core components, it is possible to construct a one-factor model, because background theory and previous studies have shown that the core components are partly overlapping (having cross-correlations) and exist in a somewhat hierarchical relationship with each other^3,21^.

The challenge in constructing a short, comprehensive, and generic health literacy instrument lies in the need to adequately take into account the complex and multidimensional nature of health literacy. Interpretative clarity of the model is essential, meaning that one must go beyond modifying every detail in pursuit of statistical adequacy (thus seeking a balance between content validity and structural validity). As there was only a small difference in explanatory power between the country-specific models and the total sample model, the items selected for the final model can be considered to form a sound and content-valid measure of health literacy, regardless of the country in question.

There was some variation between countries regarding the items measuring theoretical knowledge, critical thinking, and citizenship. The items finally selected for the model were ‘I have good information about health’, ‘I can compare health-related information from different sources’ and ‘I can judge how my own actions affect the surrounding natural environment’. Note that regarding the items that were not selected for the model, the respondents had to assess their ability to give examples on how to promote health (theoretical knowledge), decide whether health information is right or wrong (critical thinking), and give ideas on how to promote health in the person's immediate surroundings, such as the nearby place or family (citizenship). These items were selected for the country-specific model in some countries, but not in others. One reason for this may be that young people from different countries may perceive their skills in the tasks required to produce differently. Items measuring practical knowledge and self-awareness (‘When necessary, I find health-related information that is easy for me to understand’ and ‘I can judge how my own actions affect the surrounding natural environment’) were found appropriate for the model in almost every country surveyed. These items may require less interpretation and be slightly less cognitively challenging than their alternative items, and they could therefore be selected for the model in a relatively unequivocal way.

For descriptive purposes, the health literacy levels were classified into three groups (low, moderate, high) based on cut-off scores. Inspection of the response distributions showed that the short HLSAC-5 instrument performed logically in the classification of health literacy levels, i.e. in the same way as the original HLSAC instrument. However, there were small differences between the two scales; thus, when HLSAC-5 was used the proportion of respondents with low and moderate health literacy was slightly smaller, and the proportion of respondents with high health literacy somewhat greater. This may be because the selected smaller number of items approached health literacy in a more limited way, thus placing greater emphasis on the single competency as expressed in a single item.

Examination of the structural validity provides evidence on the construct validity of the instrument^46,47^. The structural validity of the HLSAC-5 was analysed via CFA, i.e. with the model-fit parameters indicating how the data fitted into the assumed factor structure. This kind of structural validation is a strong method to examine the validity of an instrument when (as was the case in this study) the instrument structure is specified a priori, and when the contextualization is based on evidence from previous research^47^. The HLSAC-5 instrument’s overall goodness and sufficiency were at a good level, and the instrument showed an excellent fit with the data in all the participating countries. This was a notable result, especially considering the large amount of data involved^39,48,49^.

The reliability of HLSAC-5 was inspected. Both item reliability and scale reliability were at an adequate level. The factor loadings and reliability coefficients (R^2^) indicated that the items were reliable measures of a latent variable (health literacy). The internal consistency reliability varied to some extent between countries, but was at a high level in the total sample, exhibiting a Cronbach alpha of 0.80. This means that the items measured the same construct. The high α-value is also noteworthy, bearing in mind that the number of items included in the instrument has an impact on the value of Cronbach’s alpha coefficient, with a low number of items reducing the α-value^50^. Despite this, the HLSAC-5 instrument with five items demonstrated a high level of internal consistency.

Regarding measurement invariance, configural and metric invariance was established across genders, ages, and countries. Scalar invariance was achieved for age and gender groups, but not between countries. This indicated that the structure of the instrument was similar across countries and that it properly measured the same phenomenon in each country, but that there were differences in the levels of health literacy between countries. The establishment of configural, metric, and partial scalar invariance, together with the excellent fit of the model to the data, indicates that partial invariance holds, and provides a sufficient condition for comparing mean values between countries^51,52^. This can be regarded as a good result, considering the number of countries involved and the size of the sample. One should note the well-known difficulty of achieving full measurement invariance in most empirical studies^53–55^; in particular, scalar invariance has been described as an unachievable ideal that can only be approximated in practice^53^. There can be several reasons why full measurement invariance is not often met in large cross-national studies such as the present study. The original HLSAC instrument, which was used as a starting point for the study, was in the English language, with subsequent translation into the language of the target countries (translation–back translation). Even if the translation process was carried out to a high standard, minor differences of interpretation may remain, and together with possible cultural differences, the meanings of the response options or the items may involve slightly different connotations. There may also be other explanations: for example, participants' understanding of the content of the items can vary, as may familiarity of the participants with the response format, or the extent to which respondents give socially acceptable answers^56,57^.

There is no gold standard scale for measuring children’s and adolescents’ health literacy, and hence no absolute criterion for assessing the validity of the HLSAC-5 instrument. Here, criterion validity was assessed in relation to self-rated health, in line with the notion of defining ‘the extent to which a construct [here, health literacy] relates to another construct that it should theoretically be related to’^20^. As measured with the HLSAC-5 instrument, health literacy was associated with differences in self-rated health in each participant country. On average, the short instrument explained 7% of the variance in self-rated health, which is similar to the results on the original 10-item HLSAC instrument^22^. Paakkari et al.^22^ discuss what coefficient of determination would be sufficient to indicate an important or critical factor for self-rated health. In line with DeSalvo et al.^24^, they emphasize that self-rated health is a robust predictor of mortality, and argue that ‘any factor that contributes to a decrease in health disparities is important, including health literacy’. It should also be noted that according to previous studies, health literacy explains more of adolescents’ self-rated health than, for example, family affluence, gender, age, school achievement, or educational orientation^8,22^, which are often considered to be valid explanatory factors.

This study was based on a previously-developed HLSAC instrument. This limited the choice of items and precluded the possibility of developing a completely new health literacy instrument. It should also be noted that the conceptual framework behind the HLSAC-5 tool is only one possible way to conceptualize health literacy. In using the instrument, it will be important to know the framework that it is based on, the purposes and contexts appropriate to its use, and the advantages or limitations thus implied. Even a good health literacy instrument can give biased results if used in the wrong context. The HLSAC-5 instrument is by nature a comprehensive and generic tool, based on a relatively broad notion of health literacy. It can provide a good overview of health literacy, but domain-specific instruments may be capable of providing a more focused picture of health literacy in a specific context. Note also that the current HLSAC-5 instrument has been validated for 13- and 15-year-olds; hence, further research is needed on the applicability of the instrument to both younger and older age groups.

Health literacy has been shown to be a relevant indicator of and contributor to adolescents’ health and wellbeing, highlighting the need to research health literacy across countries, and to develop appropriate measurement tools for this purpose. The present study indicated that HLSAC-5 is a valid and reliable instrument for use in a cross-national context. Its brevity also makes it applicable as a component of large-scale surveys in which a range of phenomena are examined at the same time.

## Methods

### Study design and participants

Data from the HBSC study were used. The HBSC study is an international World Health Organization collaborative cross-sectional study. It is conducted every four years in a growing network of countries and regions within the World Health Organization European Zone, and in North America^58,59^. Following the common international HBSC research protocol, nationally representative data sets were ensured using random cluster sampling with the school as the primary sampling unit^60,61^. Within each school, one class was randomly selected. All countries in the HBSC study comply with the required ethical and data-gathering standards. Students answered the questionnaire voluntarily and anonymously during school hours via an electronic or paper–pencil survey^60^.

For the present study, a population-based cross-sectional design was used. The study covered those countries that included the measure of health literacy for both 13- and 15-year-olds in the 2017/2018 survey. The total sample consisted of 25,425 adolescents from seven countries (Table 7).

**Table 7:** Sample sizes by country, and proportions in the sample for demographic variables and self-rated health.

Country	Total n	Gender boys (%)	Age category 13-year-old (%)	Self-rated health (%)
Finland	2194	49.7	51.0	Excellent 19.5 Good 61.1 Fair 16.4 Poor 3.0
Estonia	3147	50.1	51.0	Excellent 33.0 Good 50.8 Fair 15.0 Poor 1.2
Poland	3507	51.7	49.2	Excellent 18.3 Good 65.0 Fair 13.9 Poor 2.8
Czechia	7768	50.5	50.9	Excellent 22.7 Good 62.3 Fair 13.7 Poor 1.3
Belgium(Fl.)	2688	50.0	45.7	Excellent 23.2 Good 60.1 Fair 15.3 Poor 1.4
Slovakia	3199	47.4	59.6	Excellent 26.3 Good 60.3 Fair 11.8 Poor 1.6
Germany	2922	46.2	48.1	Excellent 33.9 Good 53.7 Fair 11.3 Poor 1.1

### Ethical approval and consent for participation

The study was conducted according to the guidelines of the Declaration of Helsinki, and it obtained ethical approval from the University of Jyväskylä Ethical Committee. The school principals gave school-level approval. Participation was anonymous and voluntary. Informed consent was obtained from all subjects involved in the study as well as from their parents/guardians.

### Measures

#### Health literacy

The HLSAC instrument^21^ was used to measure the adolescents’ subjective (self-reported, perceived) health literacy. The validated 10-item instrument contains two items from each of five previously-identified core components, namely theoretical knowledge, practical knowledge, critical thinking, self-awareness, citizenship^3^. Respondents evaluated the 10 items starting with ‘I am confident that…’ on a 4-point scale (1 = not at all true, 2 = not completely true, 3 = somewhat true, 4 = absolutely true).

#### Self-rated health

Self-rated health was evaluated by a single question measuring the individual’s evaluation of their health and having four response options (1 = excellent, 2 = good, 3 = fair, 4 = poor)^62^. For the purposes of CFA the scale was reversed to have higher values indicating higher self-rated health (reversed values: 1 = poor, 2 = fair, 3 = good, 4 = excellent).

#### Demographic characteristics

Gender (1 = girl, 2 = boy) was self-reported. Age was computed based on the respondent’s month and year of birth, and the date of the survey assessment. According to the HBSC protocol, respondents are assigned to three age categories, i.e. as being of ages 11 (≥ 10.5 and ≤ 12.5), 13 (> 12.5 and ≤ 14.5), and 15 (> 14.5 and ≤ 15.5). The age categories 13 and 15 years were used in this study.

### Data analysis

First of all*, regression analysis* was used to determine the combination of five health literacy items that best predicted the original HLSAC instrument (consisting of 10 items). In the regression analyses, all the different sets of five items were tested for their predictive ability with regard to the original HLSAC. In line with the theoretical basis of the HLSAC^21^, all the 5-item sets were constructed in such a way as to contain one item from each theoretical core component included in the original HLSAC instrument. The rationale for choosing regression analysis as a tool to determine the items for the new HLSAC-5 measure was, in part, because we wanted to stay true to the original scale as far as reasonably possible, and hence achieve an explanatory power as close as possible to that of the original scale. We further theorized that closeness to the original scale would yield similar characteristics and associations to other variables (notably self-rated health) as the original HLSAC. Regression analysis was deemed a suitable tool, since it would allow us to use theoretical reasoning in choosing the restrictions that had to be in place for the items in the new HLSAC-5 measure.

Only 32 models met the condition that there should be one item from each of the five core components. In total, 32 regression models were fitted for each country in the dataset, as were 32 models for all the countries together. In all the fitted models the original 10-item HLSAC instrument was considered to be the response variable, and the candidate short HLSAC instrument the predictor. The fitted models were ranked by their adjusted R^2^ values, and the model with the highest coefficient of determination was regarded as comprising the best 5-item HLSAC instrument in terms of predicting the original HLSAC instrument.

To classify the responses into low, moderate, and high health literacy levels, sum scores were used. In the 10-item HLSAC measure, the classifications were built in the following manner: low (score 10–26), moderate (score 27–35), and high (score 36–40)^63^. For the 5-item HLSAC-5 measure, the thresholds for the levels were set by adopting a similar line of reasoning as for the longer measure: low (score 5–12), moderate (score 13–17), and high (score 18–20).

*Structural validity* was used to assess the extent to which the five selected items reflected the underlying dimension, i.e. health literacy. The structural validity was examined by confirmatory factor analysis (CFA). In the one-factor model, standardized factor loadings indicate direct structural relations between the latent factor and the item^64^. The CFA model fit was evaluated using the χ^2^-test and the following fit-indices: Tucker Lewis index (TLI), comparative fit index (CFI), standardized root mean square residual (SRMR), and RMSEA^34^. For TLI and CFI, values equal to or above 0.95 represent a good fit^48^. The RMSEA and SRMR values should be less than 0.06 and 0.08, respectively^48^.

The *reliability* of the five items was estimated by reliability coefficients (R^2^), and the internal consistency of the scale by Cronbach’s α. An alpha higher than 0.70 is regarded as indicating good internal consistency^65^.

*Measurement invariance* across gender, age, and country was determined through a multi-group analysis. We tested whether the underlying factor structure was consistent, regardless of whether the scale was used with girls or boys, with 13- or 15-year-olds, or with different countries. The models were first estimated for subgroups independently. The equivalence of the factor structure parameters was then tested in a hierarchical order (for configural, metric, and scalar invariance) as suggested by Byrne^66^. Configural invariance, with no restrictions, was first estimated. The metric invariance was tested by constraining the factor loadings to be equal across groups. Scalar invariance was examined by constraining the item intercepts to be equal across groups. Measurement invariance was established when the restrictions decreased CFI by not more than 0.010 and increased RMSEA by not more than 0.015, relative to the prior model^67,68^.

*Criterion validity* refers to the extent to which a construct relates to another construct that it should theoretically be related to Ref.^20^. As better self-rated health has been found to be moderately associated with higher health literacy in numerous studies^8,22,25–28^, a positive association between these two factors was used as an indicator of appropriate criterion validity. In this study, structural equation modelling was used to analyse the association between health literacy and self-rated health; this was done by combining CFA and regression analysis within the same model.

All the analyses were conducted with the Mplus 7.0 statistical package^69^, except for regression analyses predicting the original HLSAC instrument with the proposed HLSAC-5 instruments, which were performed with the statistical analysis software R^70^. The CFA parameters were estimated using the maximum likelihood robust estimation method (MLR). Cases with missing values were included in the analyses and treated with the missing-at-random data procedure in Mplus.

## Data Availability

The data were provided by the HBSC Data Management Centre at the University of Bergen, Norway, to which requests for data should be submitted (dmc@hbsc.org).
